# Validation of the Chinese version of the Valued Living Questionnaire among Chinese patients with psychiatric illness

**DOI:** 10.3389/fpsyt.2026.1760394

**Published:** 2026-08-06

**Authors:** Wenjun Song, Jing Li, Che Mohd Nasril Che Mohd Nassir, Mohammad Farris Iman Leong Bin Abdullah, Mohd Salami Ibrahim

**Affiliations:** 1 The Second Affiliated Hospital of Zunyi Medical University, Zunyi, Guizhou, China; 2 The Second Affiliated Hospital of Henan Medical University, Xinxiang, Henan, China; 3 Department of Anatomy and Physiology, Faculty of Medicine, Universiti Sultan Zainal Abidin, Kuala Terengganu, Terengganu, Malaysia; 4 Department of Psychiatry and Mental Health, Faculty of Medicine, Universiti Sultan Zainal Abidin, Kuala Terengganu, Terengganu, Malaysia; 5 Department of Health Professional Education, Faculty of Medicine, Universiti Sultan Zainal Abidin, Kuala Terengganu, Terengganu, Malaysia

**Keywords:** China, psychiatric illness, reliability, translation, validity, valued living questionnaire

## Abstract

**Background:**

Valued living is a core process in Acceptance and Commitment Therapy (ACT), and the Valued Living Questionnaire (VLQ) is widely used to assess this construct. However, the psychometric properties of the Chinese version of the VLQ (VLQ-C) have not been established. This study aimed to translate the VLQ into Chinese and evaluate its psychometric properties using complementary Item Response Theory (IRT) and Classical Test Theory (CTT) approaches.

**Methods:**

The VLQ was translated and culturally adapted in accordance with international guidelines. Face and content validity were established prior to administration among 300 Chinese-speaking patients with psychiatric illness. Reliability was assessed using ordinal Cronbach’s alpha, McDonald’s omega, and test–retest reliability. Convergent and discriminant validity were evaluated using the Chinese versions of the Acceptance and Action Questionnaire-II (AAQ-II-C) and Generalized Anxiety Disorder-7 (GAD-7-C), respectively. Rasch Partial Credit Model (PCM) analysis was performed to evaluate category functioning, item fit, reliability, separation, and targeting. Polychoric confirmatory factor analysis (CFA) using robust weighted least squares mean, and variance adjusted estimation (WLSMV) was conducted to compare competing factorial structures.

**Results:**

The VLQ-C showed excellent face/content validity. Internal consistency was good to excellent (α = 0.834–0.935; ω = 0.834–0.934), and test–retest reliability was excellent (ICC = 0.826–0.865). Convergent validity was supported by moderate correlations with psychological flexibility (r = 0.501–0.680); discriminant validity by weak negative correlations with anxiety (r = –0.132 to –0.282). Rasch PCM favored a 5-category over the original 10-category format, with better category functioning, item fit, targeting, and model performance. Polychoric CFA supported a two-factor model with correlated residuals as the best-fitting structure.

**Conclusion:**

The VLQ-C shows promising psychometric properties for assessing valued living in Chinese-speaking psychiatric patients. Independent validation in clinical and community samples is recommended prior to broader use.

## 1 Introduction

As a “third-wave cognitive behavioral therapy,” Acceptance and Commitment Therapy (ACT) goes beyond merely alleviating suffering, placing a greater emphasis on “achieving psychological well-being through living out personal values.” “Valued living” is precisely the core process within ACT that connects “values cognition” with “behavioral change” (1, 2). Defined within the ACT framework as “freely chosen, verbally constructed patterns of behavior that align with intrinsic reinforcers” (3), valued living emphasizes not just reducing psychological distress, but guiding individuals to engage in actions consistent with their core values, such as family, work, or spirituality, to enhance life meaning and quality (4). To operationalize and measure this construct, Wilson and colleagues developed the VLQ, a brief, theoretically grounded tool that assesses two key facets of valued living: the importance of 10 core life domains and an individual’s consistency in acting in line with those valued domains (3). The VLQ has been translated and validated in Spanish and Japanese (5, 6). In addition, another instrument, which is also self-reported and used to measure valued living, is the Valuing Questionnaire (VQ), which consists of 10 items and two factors (7). However, the VQ lacks evidence regarding its psychometric properties and hence has not fully evidenced its validity to measure valued living.

Basically, the lack of a validated Chinese measure of valued living hinders two critical goals: [1] advancing cross-cultural ACT research, which requires tools that capture cultural variations in core processes like valued living; and [2] implementing values-based interventions in Chinese clinical and community settings, where demand for ACT and related therapies is growing, but culturally appropriate assessment tools are scarce (1). Without a valid VLQ-C, researchers and practitioners cannot accurately assess how Chinese individuals engage with valued living, nor evaluate the effectiveness of ACT interventions targeting this construct. The original version of the VLQ was developed and validated in the American college student population (2). Nonetheless, there may be large differences between Chinese and American cultures, whereby Chinese families and extended communities are more collectivistic, placing the needs of the family or extended community beyond the self’s needs. Family harmony, obligations, and loyalty are the top priorities in Chinese culture. Friends and colleagues are treated as extended family, and the Chinese value loyalty and harmony with co-workers. Moreover, Chinese people may not be as expressive as Americans, so any psychological distress may not be communicated to others and may be presented as somatic complaints (8, 9). Consequently, due to these cultural differences, the psychometric properties of the Chinese and the original English versions of the VLQ may also be different.

There are certain unresolved limitations in the use of the VLQ as a self-administered instrument, such that it was designed for responses to be interpreted qualitatively and not to be scored as an overall value of living degree. The instruction and structure of the instrument do not prevent the responders from answering with a score of 10 for all the items, and hence the measurement is complicated by social desirability (2). To address these gaps, the present study aimed to adapt the original English version of the VLQ into a Chinese version and systematically examine its psychometric properties among Chinese individuals with psychiatric illness. Specifically, this study aimed to: [1] translate and culturally adapt the original English version of the Valued Living Questionnaire (VLQ) into Chinese and establish its face and content validity; [2] evaluate the measurement properties of the Chinese version of the VLQ (VLQ-C), including reliability, convergent validity, and discriminant validity; and [3] investigate the structural validity of the VLQ-C using complementary Item Response Theory and Classical Test Theory frameworks, including Rasch Partial Credit Model analysis, polychoric confirmatory factor analysis, and ordinal reliability estimation. By validating the VLQ-C, this study seeks to provide a culturally appropriate tool for assessing valued living in China while contributing to a broader understanding of how cultural contexts shape the measurement of ACT core processes.

## 2 Methods

This study was divided into two phases: Phase I, which involved translation and back-translation, and the establishment of face and content validity of the VLQ-C; and Phase II, which involved validation of the final draft of the VLQ-C.

### 2.1 Phase I

Phase I of the study commenced from March to April 2025.

#### 2.1.1 Translation and back translation of the Chinese version of the Valued Living Questionnaire and Assessment of Content Validity

This study followed the procedures according to the ITC Guidelines for Translating and Adapting Tests (Second Edition) (10). Two independent language specialists, bilingual native Chinese speakers from the School of Foreign Languages at Henan Medical University, translated the VLQ’s original English edition into Chinese. Subsequently, a bilingual language expert from the same institution, fluent in English and not having read the original version of the VLQ, performed a back-translation from Chinese into English. To establish the content validity of the translated Chinese version, a team of experts made up of two psychiatrists, two psychologists, and two community health professionals evaluated the translation and back-translation drafts of the VLQ (the two community health professionals were included in the panel of experts because their area of research also focuses on mental health in the community).

The selection criteria for the panel of experts were: [1] engaged in mental health patients for at least 5 years after completion of a postgraduate qualification, [2] academic qualification with a Master of Medicine or PhD, and [3] voluntary participation in the study. The panel’s experts were individually asked to assess the relevance of each item to the VLQ’s domains using the available answer choices. The answer options for each item were: [1] the item is not relevant to the measured domain, [2] the item is partially relevant to the measured domain, [3] the item is relevant to the measured domain, and [4] the item is very relevant to the measured domain. Those who rated an item with answer options [3] and [4] were given a score of 1, while those who placed an item with answer options [1] and [2] were assigned a score of 0. Dividing the number of raters in agreement by the total number of raters will produce the item-level content validity index (I-CVI). An I-CVI value of ≥ 0.83 was acceptable (11).

An item’s universal agreement (UA) is equal to 1 if all the experts in the panel agree that the item is either “relevant to the domain measured” or “very relevant to the domain measured”; otherwise, its UA is scored 0. The scale-level content validity index (S-CVI/UA) was calculated as the sum of UA divided by the total number of items on the VLQ; a value ≥ 0.8 is considered high. At the same time, I-CVI values are summed and divided by the total number of VLQ items to obtain the average scale-level content validity index (S-CVI/Ave), with a value ≥ 0.9 deemed high (11). After the panel of experts examined the drafted translations and back-translated copies of the VLQ, the drafted VLQ-C was constructed.

#### 2.1.2 Pilot study to assess the face validity of the Chinese version of the Valued Living Questionnaire

Then, the draft of the VLQ-C was administered to 30 subjects who were recruited from the source population, such as psychiatric patients registered for follow-up in the targeted centers (10 patients had major depressive disorder, 10 had anxiety disorder, and another 10 had major depressive disorder with comorbid anxiety disorder; all on treatment). All 30 subjects were interviewed by the research team regarding: [1] clarity, [2] comprehensibility, [3] duration of administration, and [4] semantic quality of the instructions, items, and response options. The subjects were also asked to score each item based on the following response options: [1] the item is not clear and understandable, [2] the item is partially clear and understandable, [3] the item is clear and understandable, and [4] the item is very clear and understandable. Those who answered the response options “the item is clear and understandable” and “the item is very clear and understandable” were assigned 1 point or as agreed.

The item-level face validity index (I-FVI) was computed by dividing the number of agreed items in the tool (items rated [3] or [4]) by the number of raters. The average scale-level face validity index (S-FVI/Ave) is the sum of the I-FVI scores divided by the number of items. Then, universal agreement (UA) was rated as 1 if all raters scored the item with response options [3] or [4]; otherwise, UA was 0. Hence, the average UA scores across all items (S-FVI/UA) were computed by the sum of all UA scores divided by the total number of items. The acceptable score for I-FVI was ≥ 0.83, while for S-FVI/Ave was ≥ 0.9 and for S-FVI/Ave was ≥ 0.8 (12). If there were any issues in the instructions, items, or response options in the tool, the VLQ-C will be reviewed again by the expert panels before another round of assessment with the pilot study.

### 2.2 Phase II

#### 2.2.1 Study design and study sample

This validation study commenced from April to August 2025. The study was approved by the Human Research Ethics Committee of Xinxiang Medical University (code: XYEFYLL-(Scientific Research)-2025-39) and abides by the ethical principles of the Declaration of Helsinki, 1964, and its subsequent amendments. The target sample size was established to meet the requirements for both Rasch analysis and confirmatory factor analysis (CFA), which are the primary psychometric validation methods used in this study. For the Rasch Partial Credit Model (PCM) analysis, we aimed for a minimum sample size of 200 participants, as this size is generally considered sufficient for obtaining stable item calibration estimates within approximately ±0.3 logits (13, 14).

Similarly, a minimum sample size of 200 participants was also targeted for the CFA. The literature on structural equation modeling commonly indicates that a sample size of at least 200 is adequate for CFA, whereas smaller samples (100–150) are more likely to encounter estimation problems, nonconvergence, and unstable parameter estimates (15).

Therefore, the minimum required sample size for this study was set at 200 participants to meet the standards for both Rasch PCM and CFA. To account for an anticipated attrition rate of 30%, the final target sample size was increased to 286 participants.

Subject recruitment for the study was conducted using a consecutive sampling approach. The research team approached patients with psychiatric illness who were registered for treatment at the Second Affiliated Hospital of Xinxiang Medical University and the Third People’s Hospital of Henan Province, China. These hospitals are tertiary referral centers for psychiatric patients in Henan Province, China, and the Second Affiliated Hospital of Xinxiang Medical University is the only mental institution in Henan Province. There are approximately 4,000 to 5,000 psychiatric patients registered for treatment at the Second Affiliated Hospital of Xinxiang Medical University, which also receives referrals from neighboring provinces, such as Shanxi, Shaanxi, Hebei, Shandong, and Anhui. The Third People’s Hospital of Henan Province has about 1,000 to 2,000 psychiatric patients registered for treatment.

The potential subjects were screened against the study’s eligibility criteria. The inclusion criteria were: [1] patients with any mental illness with a diagnosis confirmed by screening with the Mini International Neuropsychiatric Interview performed by the psychiatrist in the research team (psychiatric patients were selected as the study sample because mental illness could alter the capability of patients to experience joy, meaning, and perception of life value, which enables the research team to evaluate how well the VLQ measures these changes; and furthermore, one of the main uses of the VLQ is to measure the effects of psychosocial interventions on perception of the value of life in people with mental illness), [2] those aged 18 years and above, [3] those who could read and write in Chinese, and [4] those who were cognitively sound to answer questionnaires (the Mini-Mental State Examination was used by the research team to screen patients, and a score of at least 25 was defined as cognitively sound). The exclusion criteria were aggressive behaviors that hindered participants’ ability to participate in the study. Participants in the research must fulfill all inclusion criteria and meet all exclusion criteria to be invited. The invited subjects were informed about the purpose of the study, the study procedures, the risks and benefits of participating, the safety procedures for data storage, and the anonymity of personal information before they signed informed consent and enrolled in the study.

The patients and the general public were not involved in the design of the study or the publication of the study findings.

#### 2.2.2 Data collection

All participants were administered the socio-demographic and clinical characteristics questionnaire, the VLQ-C, the AAQ-II-C to assess convergent validity, and the Chinese version of the GAD-7 to assess discriminant validity.

Measures:

(1) Valued Living Questionnaire (VLQ):

The VLQ, a self-report measure developed based on ACT theory, is used to assess participants’ level of valued living. It comprises two core subscales—the Importance Scale (measuring the subjective importance of different life domains to participants) and the Consistency Scale (measuring the consistency between participants’ behaviors across various life domains and their own values) —with items scored on a 1–10 Likert scale. The Valued Living Composite Score, calculated by multiplying the two subscale scores, reflects the overall level of valued living. It has favorable psychometric properties, with the composite score having acceptable-to-good internal consistency (α: 0.65, 0.74) and good test-retest reliability (0.75) (2).

(2) Acceptance and Action Questionnaire II (AAQ-II):

The AAQ-II is a self-reported instrument that measures the degree of psychological inflexibility. It consists of 7 items and is unidimensional. Each item is assessed on a Likert scale from 0 to 7, with a total score from 0 to 49. Psychological flexibility can be reflected by inverting the total score (16). The Chinese version of the AAQ-II (AAQ-II-C) is primarily designed to accurately measure individuals’ level of psychological inflexibility in the Chinese population. The AAQ-II-C was selected as a comparator to assess the convergent validity of the VLQ-C, as there is currently no instrument that measures valued living that has been translated and validated in Chinese (17).

(3) The 7-item Generalized Anxiety Disorder Questionnaire (GAD-7):

The GAD-7-C is an adapted instrument based on the original GAD-7 (18), developed to assess the presence and severity of generalized anxiety disorder symptoms in Chinese populations. It consists of 7 items that measure the frequency of anxiety-related experiences over the past two weeks, with each item scored on a 4-point scale. Previous studies have confirmed its good psychometric properties (19).

(4) Socio-demographic and clinical characteristics questionnaire:

The socio-demographic characteristics questionnaire collected information on variables such as gender, monthly income, marital status, level of education, and diagnosis. The clinical questionnaire collected data, such as psychiatric diagnosis.

### 2.3 Data analysis

All statistical analyses were performed using R version 4.3.1 (R Foundation for Statistical Computing, Vienna, Austria), accessed via the RStudio interface (version 2023.06.1, Build 524). Descriptive statistics were used to summarize participants’ socio-demographic and clinical characteristics. Categorical variables were presented as frequencies and percentages, whereas continuous variables were reported as means and standard deviations (SD). There was no missing data.

The test–retest reliability of the VLQ-C was evaluated using the intraclass correlation coefficient (ICC) by comparing baseline scores with scores obtained two weeks later among the same participants. The total number of study participants for test-retest reliability was 100 (N = 100) and the two-way mixed effect model was applied with absolute agreement. Internal consistency reliability was assessed using Cronbach’s alpha and McDonald’s Omega. Values of ≥ 0.7 were considered acceptable (20).

Before convergent and discriminant validity were assessed, the normality of the data was tested using histograms, Q-Q plots, and the Kolmogorov–Smirnov test (where p > 0.05 indicated a normal distribution). Then, convergent validity was examined by assessing correlations between VLQ-C scores and the reverse-scored total score of the Chinese version of the Acceptance and Action Questionnaire-II (AAQ-II-C), a measure of psychological flexibility. Discriminant validity was evaluated by assessing correlations between VLQ-C scores and the total score of the Chinese version of the Generalized Anxiety Disorder-7 (GAD-7-C) questionnaire. Spearman’s rank correlation coefficient was used because the scores were not normally distributed, as indicated by the histogram, Q-Q plot, and Kolmogorov-Smirnov test with p < 0.05. Statistical significance was set at p < 0.05 (two-tailed).

Construct validity was evaluated using complementary Item Response Theory (IRT) and Classical Test Theory (CTT) approaches.

#### 2.3.1 Rasch partial credit model analysis and category functioning

For the IRT, the original 10-category response format of the VLQ-C was evaluated using the Rasch Partial Credit Model (PCM) to determine whether the response categories functioned as intended. Four alternative category structures were examined and compared: [1] the 10-category model (original scale); [2] the 8-category model, whereby categories 1 and 2 were collapsed and categories 9 and 10 were collapsed; [3] the 7-category model, whereby categories 1–2, 3–4, and 9–10 were collapsed to reduce redundancy in both extreme and lower middle categories; [4] the 5-category model, whereby every two adjacent categories were combined (1–2, 3–4, 5–6, 7–8, and 9–10).

The competing category structures were compared using threshold ordering, the Akaike Information Criterion (AIC), the Bayesian Information Criterion (BIC), and Wright maps. Attention was given to the count of disordered thresholds, indicating respondents’ inability to consistently distinguish between adjacent response categories.

#### 2.3.2 Item fit

Item fit was evaluated using infit and outfit mean square (MnSq) statistics to determine the extent to which individual items conformed to Rasch model expectations. Good item fit was considered for MnSq values between 0.5 and 1.5.

#### 2.3.3 Reliability and separation

Reliability and separation indices were calculated to assess the instrument’s precision and its ability to distinguish among different levels of the latent construct. Person reliability and person separation were used to assess the extent to which the VLQ-C could differentiate respondents with varying levels of valued living. Item reliability and item separation were used to assess the stability of item-difficulty estimates and the adequacy of the sample size for calibrating the item hierarchy. Higher reliability and separation values indicate greater measurement precision and better discrimination among persons and items.

#### 2.3.4 Targeting

Targeting was evaluated using Wright maps (person–item maps), which display the distribution of person measures and item threshold locations on a common logit scale. Wright maps were visually inspected to determine the extent to which item difficulties were appropriately matched to respondent abilities. Good targeting was considered present when the distributions of persons and items demonstrated substantial overlap, indicating that the instrument adequately measured the full range of valued living within the study sample. Evidence of floor effects, ceiling effects, or substantial gaps in item coverage was also examined.

#### 2.3.5 Confirmatory factor analysis

For the CTT approach, confirmatory factor analysis (CFA) was performed using the polychoric correlation matrix and the robust weighted least squares mean and variance adjusted (WLSMV) estimator, which is recommended for ordinal response data (21). Four factorial structures were compared: [1] One-Factor Model, [2] Standalone Two-Factor Model, [3] the original item-paired Two-Factor Model, and [4] the final item-paired Two-Factor Model with correlated residuals based on theory and modification indices.

Competing measurement models were evaluated and compared based on theoretical considerations and Rasch findings. Model fit was assessed using the Comparative Fit Index (CFI), Tucker–Lewis Index (TLI), Root Mean Square Error of Approximation (RMSEA), and Standardized Root Mean Square Residual (SRMR). Values of CFI and TLI ≥ 0.90, RMSEA ≤ 0.06, and SRMR ≤ 0.08 were considered indicative of good model fit (15).

## 3 Results

### 3.1 Phase I

#### 3.1.1 Content validity of the VLQ-C

**Table 1** provides an overview of the content validity index of the VLQ-C. All the VLQ-C items’ I-CVI values ranged between 0.83 and 1.00. The VLQ-C has an S-CVI/Ave of 0.97. Finally, the ICQ-M’s S-CVI/UA was 0.80. These findings indicated that the VLQ-C achieved good content validity.

**Table 1:** Content validity index of the Chinese version of the Valued Living Questionnaire.

Items	Number of subjects in agreement	I-CVI	UA
Item 1 (I)	6	1	1
Item 2 (I)	6	1	1
Item 3 (I)	6	1	1
Item 4 (I)	5	0.83	0
Item 5 (I)	6	1	1
Item 6 (I)	5	0.83	0
Item 7 (I)	6	1	1
Item 8 (I)	6	1	1
Item 9 (I)	6	1	1
Item 10 (I)	6	1	1
Item 1 (C)	6	1	1
Item 2 (C)	6	1	1
Item 3 (C)	6	1	1
Item 4 (C)	5	0.83	0
Item 5 (C)	6	1	1
Item 6 (C)	5	0.83	0
Item 7 (C)	6	1	1
Item 8 (C)	6	1	1
Item 9 (C)	6	1	1
Item 10 (C)	6	1	1
S-CVI/Ave		0.97	
S-CVI/UA			0.80

I, importance; C, consistency; I-CVI, item-level content validity index; UA, universal agreement; S-CVI/Ave, average scale level content validity index; S-CVI/UA, scale level content validity index.

#### 3.1.2 Face validity of the VLQ-C

Interviews with the 30 patients in the pilot study revealed that 90% of them were satisfied with the semantic quality, duration of administration, comprehensibility and clarity of the items, response choices, and instructions of the Chinese version of the VLQ.

**Table 2** summarizes the face validity index for the Chinese version of the Valued Living Questionnaire. The I-FVI scores ranged from 0.93 to 1.0. While the S-FVI/Ave score was 0.99, the S-FVI/UA score was 0.8. Hence, no further revision of the VLQ-C draft was needed. These findings revealed that the VLQ-C achieved good face validity.

**Table 2:** The face validity index for the VLQ-C.

Items	Number of subjects in agreement	I-FVI	UA
Item 1 (I)	30	1	1
Item 2 (I)	30	1	1
Item 3 (I)	30	1	1
Item 4 (I)	28	0.93	0
Item 5 (I)	30	1	1
Item 6 (I)	28	0.93	0
Item 7 (I)	30	1	1
Item 8 (I)	30	1	1
Item 9 (I)	30	1	1
Item 10 (I)	30	1	1
Item 1 (C)	30	1	1
Item 2 (C)	30	1	1
Item 3 (C)	30	1	1
Item 4 (C)	28	0.93	0
Item 5 (C)	30	1	1
Item 6 (C)	28	0.93	0
Item 7 (C)	30	1	1
Item 8 (C)	30	1	1
Item 9 (C)	30	1	1
Item 10 (C)	30	1	1
S-FVI/Ave		0.99	
S-FVI/UA			0.80

I, importance; C, consistency; I-FVI, item-level face validity index; UA, universal agreement; S-FVI/Ave, average scale-level face validity index; S-FVI/UA, scale-level face validity index.

### 3.2 Phase II

#### 3.2.1 Participants

**Table 3** summarizes the socio-demographic and clinical characteristics, the mean VLQ-C composite, and the importance and consistency scores for all participants in this study. A total of 280 participants completed this study. The mean composite, importance, and consistency VLQ scores were 136.8 (standard deviation [SD] = 42.4), 70.4 (SD = 23.7), and 66.5 (SD = 23.0), respectively.

**Table 3:** Socio-demographic and clinical characteristics, and the mean VLQ-C score of the participants.

Variables	Number of participants(n)	Percentage (%)
Gender		
Male	85	30.4
Female	195	69.6
Monthly income		
< RMB 1000	91	32.5
RMB 1000 to 6000	146	52.1
> RMB 6000	43	15.4
Education^c^		
Primary or below	122	43.6
Secondary	158	26.4
Tertiary		
Marital status:	111	39.6
Married	169	60.4
Single/divorcee/widow/widower		
Psychiatric diagnosis^d^:	126	45.0
Depression only	123	43.89
Anxiety disorder only	31	11.1
Depression with co-morbid anxiety disorder	85	30.4
Other diagnoses	195	69.6
Variables	Mean	Standard deviation
Total VLQ-C score	136.0^a^	41.3^b^
VLQ-C (Important) score	69.8^a^	21.9^b^
VLQ-C (Consistent) score	66.2^a^	23.2^b^

^a^ mean; ^b^standard deviation; ^c^primary education: Grade 1 to 6, secondary education (middle and high school): Grade 7 to 12, and tertiary education: university education from undergraduate to PhD; ^d^the psychiatric diagnoses of the participants were confirmed by the Mini International Neuropsychiatric Interview, where depression only patients were diagnosed with major depressive disorder; anxiety disorder only patients were diagnosed with generalized anxiety disorder/panic disorder/agoraphobia/social anxiety disorder; depression with co-morbid anxiety disorder patients were diagnosed with major depression disorder co-morbid with generalized anxiety disorder/panic disorder/agoraphobia/social anxiety disorder; and Other diagnoses included patients diagnosed with obsessive compulsive disorder, posttraumatic stress disorder, and attention deficit hyperactive disorder.

#### 3.2.2 Reliability of the VLQ-C

(1) Internal consistency:

The internal consistency of the composite, importance, and consistency of the Chinese version of the Valued Living Questionnaire are presented in Ordinal Cronbach’s α and McDonald’s α in **Table 4**. The VLQ-C composite, importance, and consistency scores exhibited good to excellent internal consistency with Cronbach’s α of 0.922, 0.834, and 0.935, respectively. The McDonald’s ω of the VLQ-C composite, importance, and consistency scores reported similar readings as Cronbach’s α, which were 0.920, 0.834, and 0.934, respectively.

**Table 4:** Reliability, standard error of measurement (SEM), and minimal detectable change (MDC95) of the VLQ-C.

	Test-retest reliability				Internal consistency	
Score	SD	ICC (95% CI) (N = 100)	SEM	MDC95	Cronbach’s alpha	McDonald’s omega
Composite	42.4	0.862* (0.674 to 0.905)	15.7	43.6	0.834	0.834
Importance	23.7	0.865* (0.645 to 0.897)	8.7	24.1	0.935	0.934
Consistency	23	0.826* (0.687 to 0.873)	9.6	26.7	0.922	0.920

*Statistical significance at p < 0.05.

(2) Test-retest reliability:

The test-retest reliability, importance, and consistency of the Chinese version of the Valued Living Questionnaire are presented in **Table 4**. The test-retest reliability of the VLQ-C was excellent, with intraclass correlation coefficients for the composite, importance, and consistency VLQ-C scores of 0.862 (95% CI = 0.674 to 0.905, *p* < 0.001), 0.865 (95% CI = 0.645 to 0.897, *p* < 0.001), and 0.826 (95% CI = 0.687 to 0.873, *p* < 0.001), respectively.

#### 3.2.3 Convergent validity of the VLQ-C

The data were not normally distributed; hence, we proceeded with the measurement of Spearman’s correlation coefficient. Spearman’s correlation coefficient between the composite, importance, and consistency VLQ-C scores and the reversal of the AAQ-II-C total score were 0.695 (*p* < 0.001), 0.553 (*p* < 0.001), and 0.697 (*p* < 0.001), respectively. These findings signified the good convergent validity of the VLQ-C.

#### 3.2.4 Discriminant validity of the VLQ-C

The data were normally distributed; hence, we proceeded with the measurement of Spearman’s correlation coefficient. Spearman’s correlation coefficient between the composite, importance, and consistency VLQ-C scores and the Chinese version of the 7-item Generalized Anxiety Disorder total score were -0.143 (*p* = 0.167), -0.200 (*p* = 0.015), and -0.292 (*p* = 0.012), respectively. Hence, the discriminant validity of the VLQ-C was good.

#### 3.2.5 Rasch partial credit model analysis

##### 3.2.5.1 Optimizing rating category structures

The original 10-category format showed substantial ceiling effects: all 20 items exceeded the >15% threshold (mean = 27.6%; highest = 46.4% for VLQ_10_important). Floor effects were minimal (mean = 7.2%), except for VLQ_8_consistent (15.7%) (See **Table 5**).

**Table 5:** Item fit analysis for the 5-rating scale model VLQ-C.

Item	Outfit_msq	Infit_msq	Outfit_z	Infit_z	Item discrimination	Outfit status	Infit status
vlq_1_important	0.926	0.968	-0.505	-0.362	0.634	Acceptable	Acceptable
vlq_2_important	1.311	0.979	2.248	-0.222	0.620	Acceptable	Acceptable
vlq_3_important	0.742	0.838	-1.993	-2.021	0.686	Acceptable	Acceptable
vlq_4_important	0.832	0.854	-1.718	-1.712	0.665	Acceptable	Acceptable
vlq_5_important	0.934	0.932	-0.659	-0.756	0.615	Acceptable	Acceptable
vlq_6_important	0.955	0.888	-0.422	-1.307	0.652	Acceptable	Acceptable
vlq_7_important	1.082	0.961	0.846	-0.447	0.629	Acceptable	Acceptable
vlq_8_important	2.035	1.630	8.452	6.595	0.359	Borderline; retained	Borderline; retained
vlq_9_important	1.017	1.004	0.206	0.070	0.599	Acceptable	Acceptable
vlq_10_important	0.781	0.818	-1.627	-2.014	0.650	Acceptable	Acceptable
vlq_1_consistant	1.036	0.847	0.359	-1.911	0.692	Acceptable	Acceptable
vlq_2_consistant	0.870	0.867	-1.168	-1.657	0.699	Acceptable	Acceptable
vlq_3_consistant	0.653	0.730	-3.422	-3.566	0.746	Acceptable	Acceptable
vlq_4_consistant	0.855	0.896	-1.539	-1.226	0.646	Acceptable	Acceptable
vlq_5_consistant	0.939	1.003	-0.592	0.062	0.621	Acceptable	Acceptable
vlq_6_consistant	0.868	0.919	-1.387	-0.968	0.650	Acceptable	Acceptable
vlq_7_consistant	0.905	0.929	-1.028	-0.834	0.639	Acceptable	Acceptable
vlq_8_consistant	1.531	1.400	5.071	4.476	0.430	Borderline; retained	Acceptable
vlq_9_consistant	0.875	0.929	-1.290	-0.821	0.644	Acceptable	Acceptable
vlq_10_consistant	0.947	0.904	-0.406	-1.141	0.649	Acceptable	Acceptable

Response frequencies indicated that all ten response categories were utilized across all items. However, responses are clustered predominantly within the upper categories (9–10), suggesting considerable redundancy in adjacent response options.

**Figure 1:**
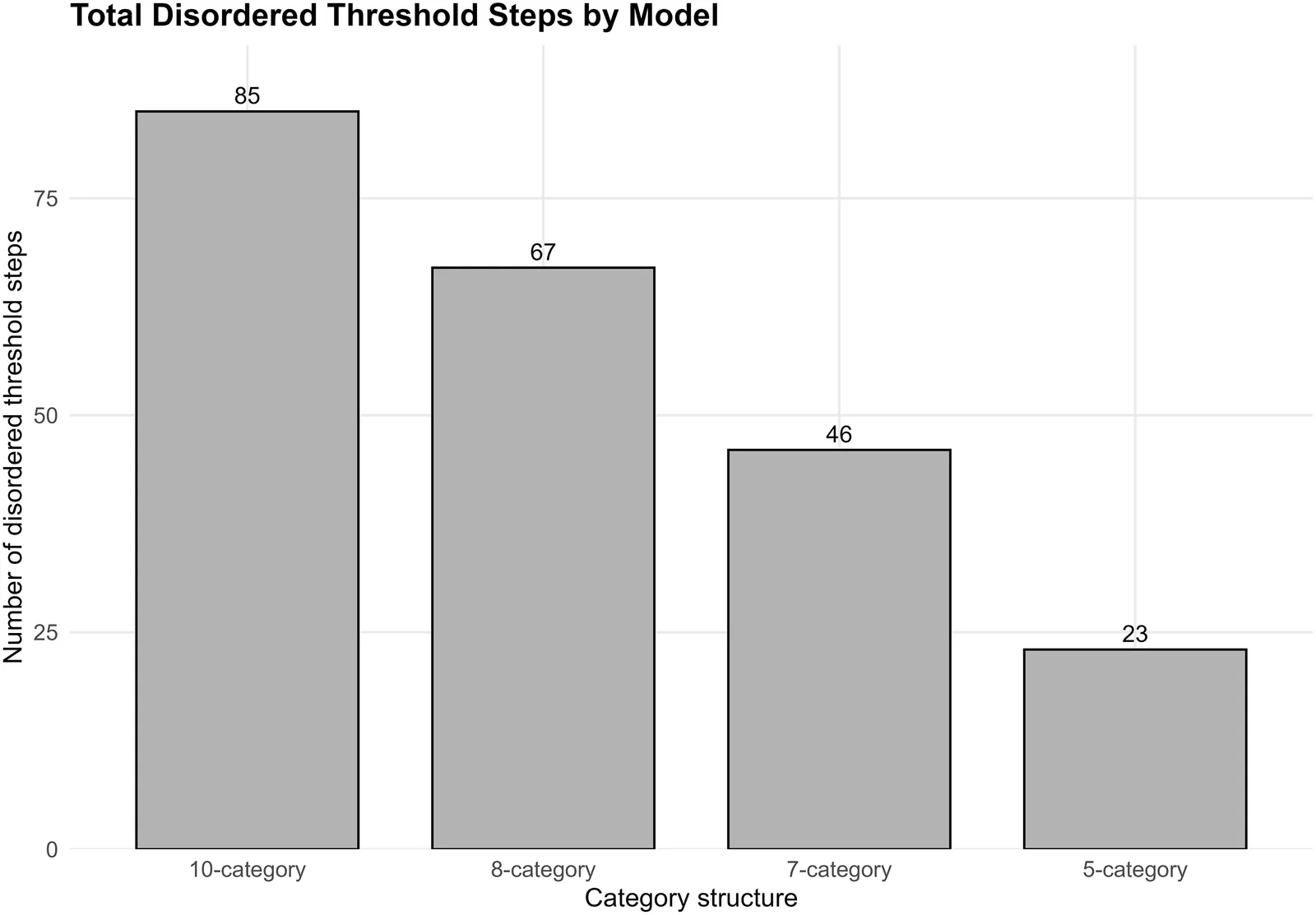
Comparison of the number of disordered thresholds between models.

Rasch PCM analysis supported this interpretation by demonstrating substantial threshold disordering in the original response structure, with 85 disordered threshold steps. Progressively collapsing adjacent categories substantially improved performance (**Figure 1**): disordered thresholds decreased from 85 (10-category) to 67 (8-category), 46 (7-category), and 23 (5-category). The 5-category model also yielded the lowest AIC and BIC values (**Table 6**).

**Table 6:** Comparison of response category models.

Model of response category	deviance	No of parameters	AIC	BIC
10-category	20441.04	201	20843.04	21573.63
8-category	17372.83	161	17694.83	18280.03
7-category	16490.74	141	16772.74	17285.25
5-category	13486.71	101	13688.71	14055.82

Although collapsing adjacent categories increased the frequency of extreme categories, the slightly larger flooring and ceiling effects were expected. It reflects the consequences of fewer available response options, rather than evidence of poorer measurement quality. The primary objective of category collapsing in Rasch PCM analysis is to improve respondents’ ability to distinguish between adjacent categories and restore ordered thresholds; the slight increase in floor or ceiling effects is acceptable.

Therefore, the 5-category model is accepted as the optimal version of the VLQ-C. We therefore propose revised wording for the response options to reflect the final scale. While the original scale ranged from 1 (Not at all important/consistent) to 10 (Extremely important/consistent), the revised 5-category version is recommended with the following anchors: 1 = Not at all important/consistent, 2 = Slightly important/consistent, 3 = Moderately important/consistent, 4 = Very important/consistent, 5 = Extremely important/consistent.

##### 3.2.5.2 Recommended new response options for the 5-category model

Given that the 5-category model represents the optimal structure for the scale, we propose revised response options to reflect this new framework. The item wording remains unchanged; only the response format is revised based on the Rasch PCM findings. Although the original scale ranged from 1 (Not at all important/consistent) to 10 (Extremely important/consistent), the validated 5-category version is recommended with the following anchors: 1 = Not at all important/consistent, 2 = Slightly important/consistent, 3 = Moderately important/consistent, 4 = Very important/consistent, 5 = Extremely important/consistent.

##### 3.2.5.3 Recommended score calculation for the 5-category model

To preserve consistency with the original VLQ scoring framework, the revised 5-category VLQ-C is recommended to retain the established scoring procedure. For each life domain, the importance item score is multiplied by the corresponding consistency item score, yielding a domain score ranging from 1 to 25. Composite valued living is then calculated by averaging the ten domain scores, with higher scores indicating greater alignment between personally important values and everyday behavior. Retaining this algorithm facilitates comparability with previous VLQ studies while leveraging the improved psychometric properties of the revised response structure.

In clinical settings, discrepancy scores – calculated by subtracting the consistency score from the importance score for each domain (e.g., importance = 5, consistency = 3; score = 2) – may also be used to identify gaps and guide intervention. Positive scores indicate a psychological gap requiring therapeutic attention. Negative scores (consistency exceeding importance) suggest external compliance or rationalization, in which patients are engaged but lack an intrinsic connection or perceived value. Such dissociations signal a need for realignment rather than behavioral escalation. Importantly, these discrepancy scores should be interpreted as clinical indicators rather than the instrument’s primary psychometric outcome.

##### 3.2.5.4 Item fit

**Figure 2:**
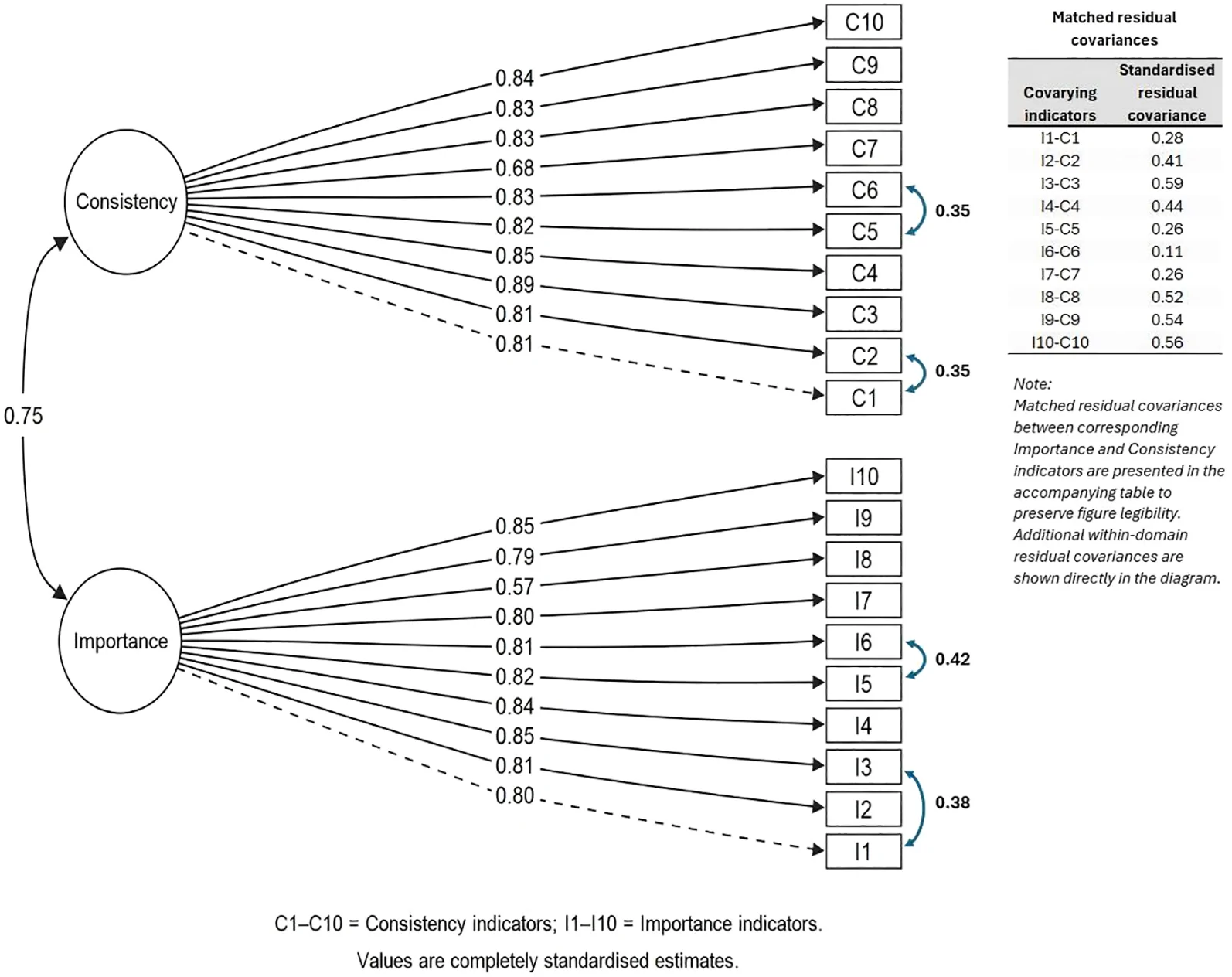
Final two-factor CFA model with standardized estimates. Within-domain residual covariances are shown; corresponding cross-domain covariances are in the adjacent table.

PCM fit statistics indicated that 18 of the 20 item components demonstrated acceptable fit (**Table 7**). Item 8 (Spirituality, Importance domain) was a misfit, with an infit mean square of error (MNSQ) of 1.630 and an outfit MnSq of 2.035 (threshold = 1.5). Because outfit statistics are particularly sensitive to unexpected extreme responses, elevated outfit values do not necessarily indicate failure of the measurement model. Furthermore, spirituality is culturally sensitive, with expected wide variation between secular and religious respondents. Individuals who are highly likely to subscribe to this value in practice are not uncommon, which explains the possibility of outlier individuals within this sample. Given its strong CFA performance below (also see **Figure 2**) and the need to preserve the 10-domain structure for content validity, Item 8 was retained for the scale’s utility and interpretation. Overall, the findings indicate that the five-category version of the VLQ demonstrates acceptable item-level fit.

**Table 7:** Item fit analysis for the 5-rating scale model VLQ-C.

Item	Outfit MNSQ	Infit MNSQ	Outfit Z-standard	Infit Z-standard	Item-total discrimination	Outfit status	Infit status
vlq_1_important	0.926	0.968	-0.505	-0.362	0.634	Acceptable	Acceptable
vlq_2_important	1.311	0.979	2.248	-0.222	0.620	Acceptable	Acceptable
vlq_3_important	0.742	0.838	-1.993	-2.021	0.686	Acceptable	Acceptable
vlq_4_important	0.832	0.854	-1.718	-1.712	0.665	Acceptable	Acceptable
vlq_5_important	0.934	0.932	-0.659	-0.756	0.615	Acceptable	Acceptable
vlq_6_important	0.955	0.888	-0.422	-1.307	0.652	Acceptable	Acceptable
vlq_7_important	1.082	0.961	0.846	-0.447	0.629	Acceptable	Acceptable
vlq_8_important	2.035	1.630	8.452	6.595	0.359	Misfitted; retained	Misfitted; retained
vlq_9_important	1.017	1.004	0.206	0.070	0.599	Acceptable	Acceptable
vlq_10_important	0.781	0.818	-1.627	-2.014	0.650	Acceptable	Acceptable
vlq_1_consistent	1.036	0.847	0.359	-1.911	0.692	Acceptable	Acceptable
vlq_2_consistent	0.870	0.867	-1.168	-1.657	0.699	Acceptable	Acceptable
vlq_3_consistent	0.653	0.730	-3.422	-3.566	0.746	Acceptable	Acceptable
vlq_4_consistent	0.855	0.896	-1.539	-1.226	0.646	Acceptable	Acceptable
vlq_5_consistent	0.939	1.003	-0.592	0.062	0.621	Acceptable	Acceptable
vlq_6_consistent	0.868	0.919	-1.387	-0.968	0.650	Acceptable	Acceptable
vlq_7_consistent	0.905	0.929	-1.028	-0.834	0.639	Acceptable	Acceptable
vlq_8_consistent	1.531	1.400	5.071	4.476	0.430	Borderline; retained	Acceptable
vlq_9_consistent	0.875	0.929	-1.290	-0.821	0.644	Acceptable	Acceptable
vlq_10_consistent	0.947	0.904	-0.406	-1.141	0.649	Acceptable	Acceptable

• Outfit (Outlier-sensitive Fit): Sensitivity to outliers—unexpected extreme responses inflate this value.

• Infit (In-pattern Fit): Performance among respondents whose ability matches item difficulty—assesses normal-condition functioning.

• MNSQ (Mean Square of Error): Noise level; 1.0 = perfect, 0.5–1.5 = acceptable for clinical use.

• Z-Standard: Deviation from model expectations; 0 = perfect, beyond ±2.0 = unexpected.

• Item-Total Discrimination: Ability to differentiate high vs. low valued living. Higher = better discrimination between respondents with different levels of valued living.

##### 3.2.5.5 Targeting

**Figure 3:**
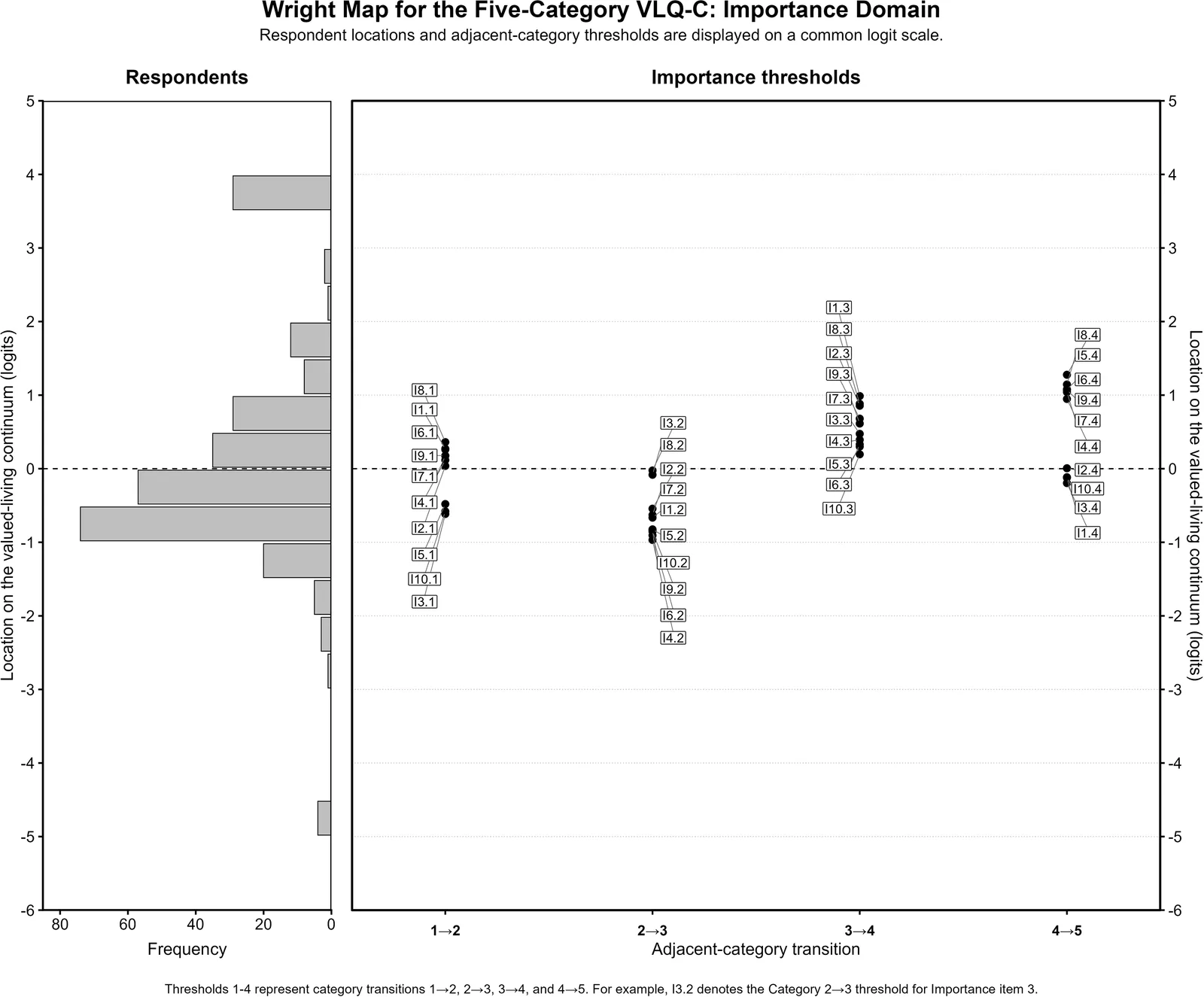
Wright map of the 5-category model of VLQ-C for the importance domain.

**Figure 4:**
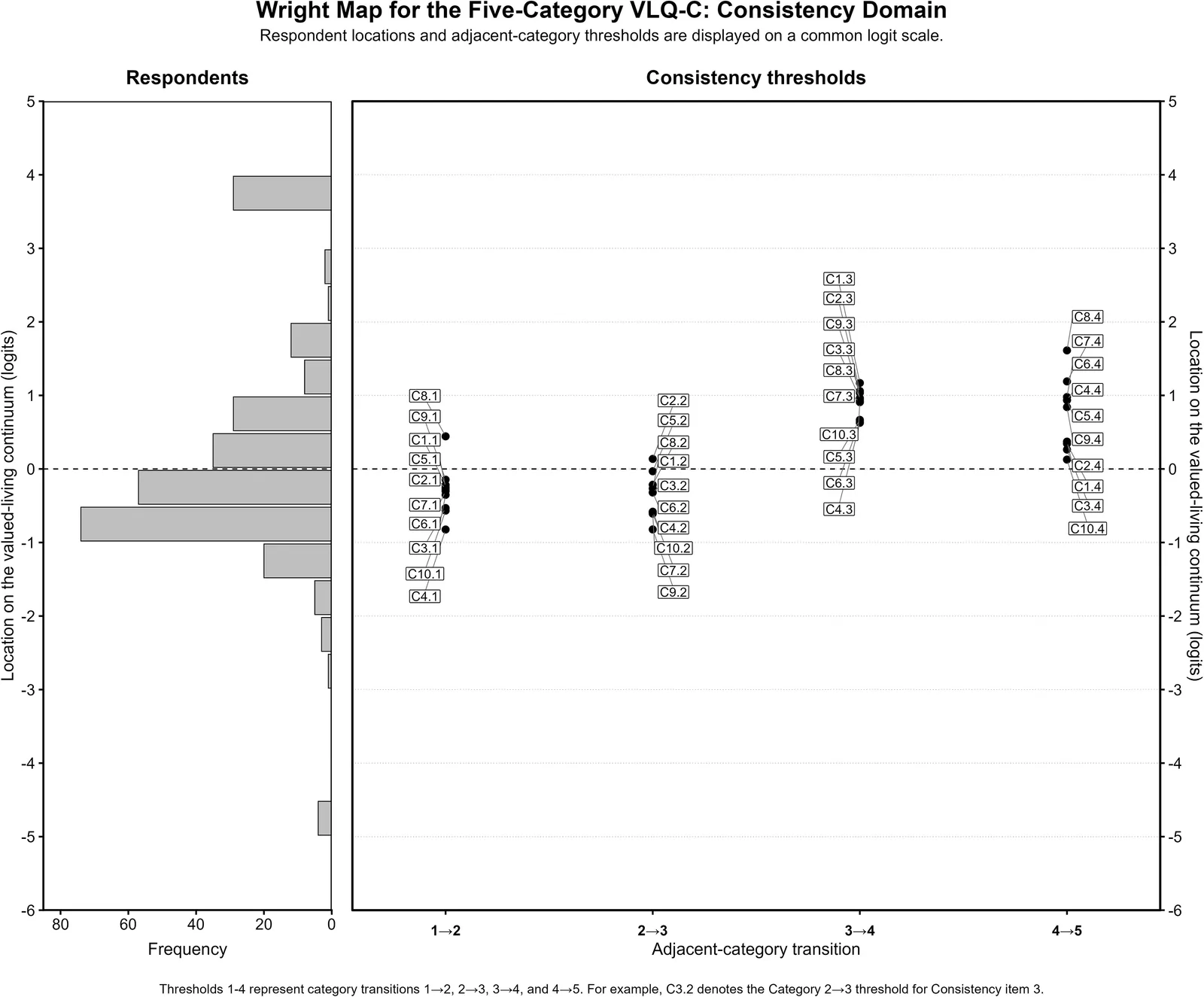
Wright map of the 5-category model of VLQ-C for the consistency domain.

**Figure 3** and **4** present the Wright person–item map, which demonstrates effective targeting of item difficulties to respondents’ latent abilities. The item thresholds are well matched to the center of the distribution, where most respondents’ measures are concentrated. Although a small percentage of respondents fall at the upper extreme, surpassing the threshold of the most challenging item, they can be considered outliers. Additionally, no significant gaps in item coverage were identified. Overall, both the statistical and visual analyses indicate good targeting, supporting the suitability of the five-category response structure for measuring values-related functioning in this sample.

##### 3.2.5.6 Differential item functioning by gender

To evaluate whether the optimized five-category VLQ-C functioned equivalently across gender groups (85 females and 195 males), differential item functioning (DIF) analysis was conducted using ordinal logistic regression. Both uniform and non-uniform DIF were assessed, and the Benjamini–Hochberg (BH) procedure was applied to control for multiple comparisons. The detailed DIF results are presented in **Table 6**.

Of the 20 item-components examined, 12 demonstrated measurement invariance across gender and showed no evidence of DIF after BH adjustment (adjusted *p* ≥ 0.05). The remaining eight item-components exhibited statistically significant gender-related DIF after correction for multiple testing (adjusted *p* < 0.05). These included five items from the Importance domain (Items 2, 3, 4, 5, and 10) and three items from the Consistency domain (Items 2, 7, and 10). Among these, Importance Item 4 showed the strongest evidence of DIF.

**Figure 5:**
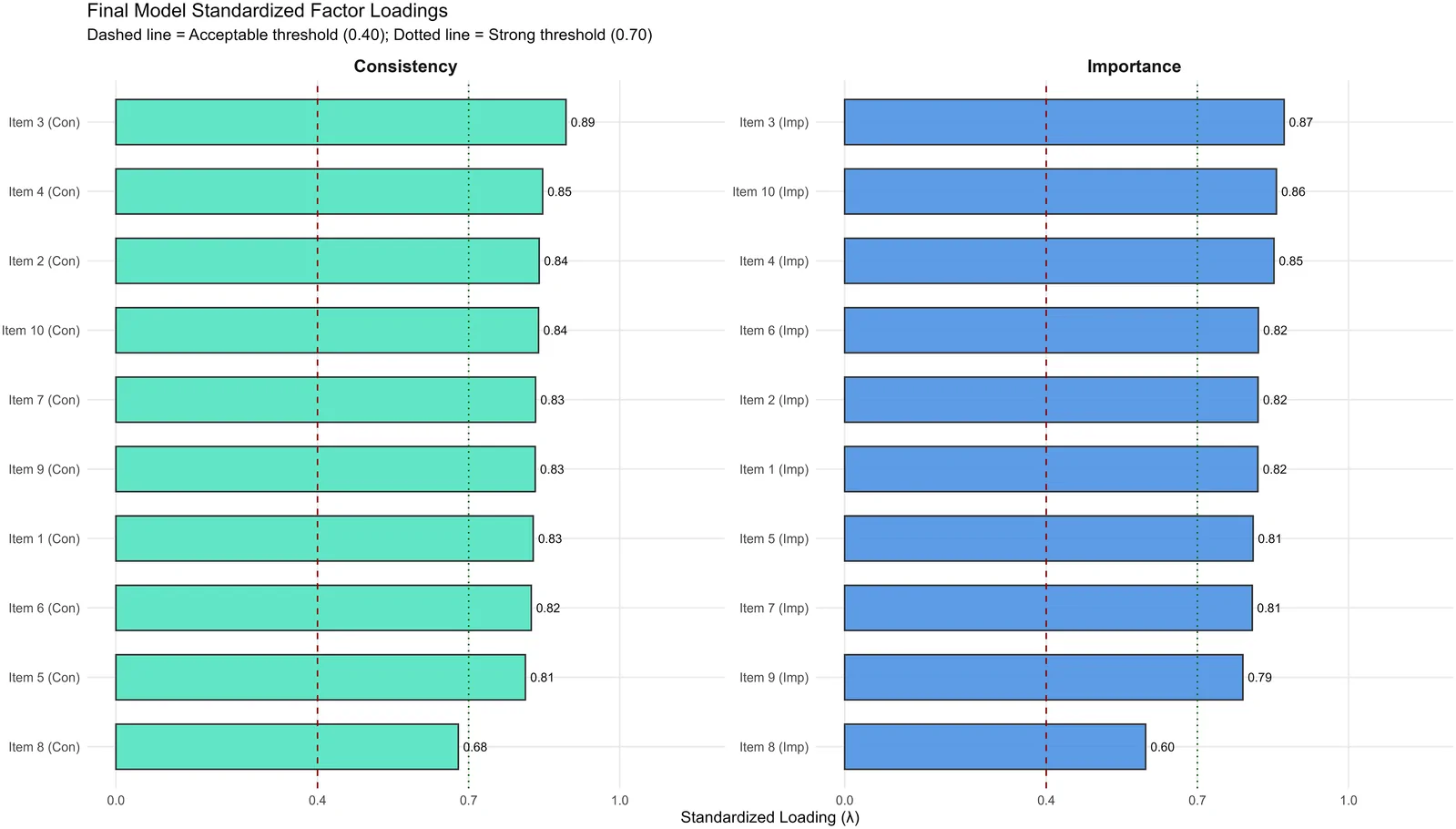
Standardized factor loading for the VLQ-C.

Although 40% of the VLQ-C items showed differential item functioning between males and females, these items were intentionally retained to preserve the comprehensive content validity and structural framework of the instrument. Statistically, all flagged items demonstrated highly acceptable psychometric performance, as indicated by the complementary confirmatory factor analysis (CFA) reported below, with solid factor loading (**Figure 5**). There are also satisfactory parameter estimates across other Rasch PCM diagnostics. All these items display robust item fit (**Table 7**), and good targeting (see **Figures 3**, **4**). More importantly, these items represent core, distinct domains essential to measuring the valued living construct within the Acceptance and Commitment Therapy (ACT) framework. Removing the flagged items will substantially undermine the assessment of this framework. Consequently, while the full 20-item scale remains psychometrically sound for broad use, these DIF findings warrant particular caution in the eight flagged items when comparing scores between male and female respondents in the Chinese population.

##### 3.2.5.7 Confirmatory factor analysis

A polychoric CFA using the WLSMV estimator was conducted to compare four competing factorial structures for the five-category VLQ-C. The sampling adequacy was confirmed by a Kaiser-Meyer-Olkin (KMO) value of 0.859 and a significant Bartlett’s test of sphericity (χ²(190) = 6208.12, p < 0.001), indicating that the sample size of 280 was excellent for this analysis.

**Figure 2** shows that the two latent factors were strongly correlated (r = 0.75), while remaining conceptually distinct. **Figure 5** presents the standardized item loadings, revealing that all items exceeded 0.7 in each domain, except for item 8, which exceeded 0.6. **Table 8** summarizes the progressive improvement in fit for the compared models following the theoretically justified incorporation of item-paired relationships with correlated residuals, and this comparison is illustrated in **Figure 2**.

**Table 8:** Comparison of model fit indices across factorial models.

Fit Index	1-Factor	2-factor Baseline	2-factor with matched item	Final Model*	Improvement
χ²/df	6.2**	5.09**	3.85**	3.33**	↓ 46%
CFI	0.903	0.924	0.95	0.961	↑ 0.058
TLI	0.892	0.915	0.941	0.952	↑ 0.060
RMSEA	0.136	0.121	0.101	0.091	↓ 0.045
SRMR	0.104	0.063	0.051	0.047	↓ 0.057

*Final Model – 2-Factor Model with Matched Item Residual Covariances and Correlated Residuals for Items 5 & 6 (Importance), Items 1 & 2 (Consistency), Items 5 & 6 (Consistency), and Items 1 & 3 (Importance).

**χ² tests were statistically significant (p < 0.001), which is common in CFA models with moderate-to-large sample sizes; therefore, model evaluation was based primarily on multiple fit indices (CFI, TLI, RMSEA, and SRMR).

Although the RMSEA remained above the predefined criterion for good model fit, the combined pattern of fit indices (CFI, TLI, SRMR, and RMSEA) indicated that this model provided the best overall fit among the competing models and was therefore retained as the preferred representation of the VLQ-C factor structure.

## 4 Discussion

This study aimed to translate the VLQ into the Chinese language and evaluate the reliability and validity of the VLQ-C. In terms of reliability, the VLQ-C registered greater internal consistency (Cronbach’s α for composite, importance and consistency of VLQ-C at 0.922, 0.834, and 0.935, respectively) compared with that of the original English version of the VLQ (Cronbach’s α for composite, importance and consistency ranged from 0.65 to 0.74, 0.79 to 0.83, and 0.58 to 0.60, respectively) (2) and the Spanish version of the VLQ (Cronbach’s α for composite, importance and consistency of VLQ-S at 0.71, 0.70, and 0.68, respectively) (5). The excellent internal consistency was confirmed for the VLQ-C as Macdonald’s α reported similar values compared with that of Cronbach’s α for the VLQ-C (Macdonald’s α for composite, importance, and consistency of the VLQ-C were 0.920, 0.834, and 0.934, respectively). Moreover, the test-retest reliability exhibited by the VLQ-C [ICC of the composite, importance and consistency of the VLQ-C were 0.862 (p < 0.001), 0.865 (p < 0.001), and 0.826 (p < 0.001), respectively] was also greater compared with that of the original English version of the VLQ (ICC of the composite, importance and consistency were 0.75, 0.90, and 0.58, respectively) (2) and comparable to the comprehension support version of the VLQ [ICC of the composite, importance and consistency of the VLQ-C were 0.82 (p < 0.001), 0.91 (p < 0.001), and 0.78 (p < 0.001), respectively] (22). In assessing the test-retest reliability of the VLQ-C, the 2-week interval between the baseline and follow-up assessments was acceptable, as no changes in the degree of valued living were expected within that period (2). The greater internal consistency and test-retest reliability registered in the VLQ-C compared with that of the original English version (2) and the translated Spanish version of the tool (5) may be attributed to differences in sample characteristics. Psychiatric patients were recruited in this study, while undergraduate university students were recruited in the validation studies of the original English and translated Spanish versions of the instrument, in which the psychiatric patients or clinical population made up a more homogeneous study sample. This point is also supported by the more comparable test-retest reliability between this study and the validation study on the comprehension support version of the VLQ, which was tested in a clinical sample (a sample with acquired brain injury) (22). Moreover, a well-executed translation may also produce items that are simpler, less ambiguous, more direct, and culturally comprehensible than the original questionnaire.

In the context of convergent validity, the composite and component scores of the VLQ-C exhibited a moderate Pearson’s correlation with the reversed total AAQ-II-C score (which measures psychological flexibility). The effectiveness of ACT in targeting psychological flexibility and ultimately the awareness of valued living and performing actions to pursue the valued living identified pinpointed that both psychological phenomena are indeed related (2). Acceptance of unpleasant thoughts and emotions (psychological flexibility) would allow a person to focus on identifying important values in life and taking necessary actions to achieve them (1). Hence, VLQ-C demonstrated good convergent validity, exhibiting a moderate correlation with the AAQ-II-C reversal score. Regarding discriminant validity, the composite and component scores of the VLQ-C exhibited only weak correlations with the total score of the GAD-7-C (which assesses the severity of anxiety symptoms), indicating that the two instruments are expected to measure different constructs. Hence, the discriminant validity of the VLQ-C was acceptable because partial discriminant validity was achieved.

Additionally, Rasch PCM analysis demonstrated that a 5-category response structure emerged as the most psychometrically defensible response structure, with acceptable item fit, reliability, separation, and targeting. Unlike classical test theory, Rasch analysis enables direct evaluation of category functioning by examining the ordering of response thresholds along the latent trait continuum. The original 10-category scale exhibited substantial threshold disordering, suggesting that respondents had difficulty consistently distinguishing between adjacent response options. Another notable finding was the pronounced ceiling effects of the original 10-category VLQ-C items. **Table 5** indicated that more than 15% of respondents chose the highest response category for all items. Theoretically, this finding suggests limited discrimination at the extreme end of the scale. The findings suggest that respondents may not meaningfully differentiate among the ten response options when evaluating valued living.

While the revised 5-category format resulted in marginally higher floor and ceiling percentages—an anticipated consequence of category reduction—this trade-off is outweighed by substantial psychometric gains. Progressive collapsing of categories resulted in improved threshold ordering and overall model performance, with **Figure 1** illustrating that the 5-category structure produced the fewest disordered thresholds and the most favorable fit statistics. The Wright maps (**Figures 3**, **4**) further demonstrate that the five-category response format provides good targeting across both domains, with item thresholds well matched to respondents’ valued-living levels. These findings suggest that the five-category structure adequately captures variation in the construct while reducing category redundancy and improving measurement efficiency. A more parsimonious five-category structure therefore appears sufficient to capture variation in the construct while reducing cognitive burden and improving measurement efficiency. Accordingly, the final VLQ-C validated in this study is recommended to adopt the 5-category response format, with the following anchors: 1 = Not at all important/consistent, 2 = Slightly important/consistent, 3 = Moderately important/consistent, 4 = Very important/consistent, 5 = Extremely important/consistent.

Beyond category optimization, five items from the Importance domain and three items from the Consistency domain demonstrate statistically significant gender-related DIF after BH correction (**Table 9**). The results suggest that these items may not function equivalently across male and female respondents. Future research with a larger, more balanced sample size may be conducted to determine whether specific gender calibration is necessary and meaningful for understanding and relevance to policies and practices. Nevertheless, most items (12 of 20) in this Chinese translation of VLQ demonstrated consistent measurement across gender, supporting the overall stability of the 5-category VLQ-C. Furthermore, the DIF-flagged items were retained because they contributed meaningfully to content coverage and demonstrated acceptable psychometric performance in the structural and Rasch analyses.

**Table 9:** Differential item functioning (DIF) analysis of the VLQ-C items by gender (85 female, 195 male).

Item	No. of categories	Uniform DIF p-value	Non-uniform DIF p-value	Total DIF p-value	BH-adjusted p-value	DIF status
VLQ-1 Importance	5	0.111	0.1819	0.1152	0.256	No DIF
VLQ-2 Importance	4	0.0202	0.0064	0.0016	0.0046	DIF after BH correction
VLQ-3 Importance	5	0.0035	0.003	0.0002	0.001	DIF after BH correction
VLQ-4 Importance	4	0.345	0.0001	0.0001	0.0001	DIF after BH correction
VLQ-5 Importance	4	0.0686	0.0001	0.0001	0.001	DIF after BH correction
VLQ-6 Importance	5	0.1874	0.2065	0.1888	0.3776	No DIF
VLQ-7 Importance	5	0.712	0.4891	0.7354	0.8416	No DIF
VLQ-8 Importance	5	0.5024	0.829	0.7802	0.8416	No DIF
VLQ-9 Importance	5	0.3282	0.1574	0.2282	0.4149	No DIF
VLQ-10 Importance	4	0.0019	0.0077	0.0002	0.001	DIF after BH correction
VLQ-1 Consistency	5	0.8342	0.1246	0.3008	0.4455	No DIF
VLQ-2 Consistency	5	0.0568	0.0015	0.0011	0.0037	DIF after BH correction
VLQ-3 Consistency	5	0.5092	0.185	0.3341	0.4455	No DIF
VLQ-4 Consistency	5	0.2548	0.5438	0.4348	0.5435	No DIF
VLQ-5 Consistency	5	0.1491	0.7094	0.3295	0.4455	No DIF
VLQ-6 Consistency	5	0.996	0.1368	0.3307	0.4455	No DIF
VLQ-7 Consistency	5	0.0195	0.1176	0.0192	0.048	DIF after BH correction
VLQ-8 Consistency	5	0.5336	0.8064	0.7995	0.8416	No DIF
VLQ-9 Consistency	5	0.9904	0.859	0.9843	0.9843	No DIF
VLQ-10 Consistency	5	0.0162	0.0048	0.0011	0.0037	DIF after BH correction

Additionally, 12 of the 20 items showed no significant DIF by gender. The remaining eight DIF-flagged items were retained, as their removal would compromise content coverage, and they exhibited satisfactory psychometric performance across all complementary Rasch PCM diagnostics, including item fit statistics, threshold ordering, and targeting indices. It is important to differentiate DIF from classical test theory (CTT)-based subgroup mean comparisons. DIF occurs when respondents from different groups who share identical levels of the underlying latent trait nevertheless display systematically different probabilities of endorsing specific response categories on an item. Thus, a DIF-flagged item indicates that gender moderates the interpretation or response process for that item, rather than reflecting a genuine difference in the latent trait itself. In contrast to CTT-based subgroup analyses, which combine true trait differences with measurement artefacts, DIF isolates and quantifies item-level bias. Accordingly, DIF provides complementary evidence regarding measurement invariance beyond conventional subgroup mean comparisons. DIF reveals whether the scale functions equivalently across groups, providing essential evidence for measurement invariance that simple mean comparisons cannot supply.

For example, within the Chinese cultural context, distinct gender roles regarding marriage and parenting have deeply rooted historical foundations. Traditionally, men are viewed as the primary leaders and providers for the household, whereas women are associated with core care, education, and nurturing of children and the family unit (23). Consequently, the observed DIF findings may reflect this socio-cultural reality. Even when Chinese men and women possess the exact same underlying trait level regarding the importance and consistency of their values in marriage (Item 2) and parenting (Item 3), they may process and respond to these items through their respective culturally socialized lenses. Exploring the precise mechanisms driving these variations is beyond the scope of this study and warrants future research with a dedicated and balanced sample size. However, they do not materially undermine the overall psychometric evidence supporting the VLQ-C. Instead, it offers a sophisticated layer of cultural insight, reminding researchers to apply tailored care when comparing sub-scale scores across genders. Crucially, the comprehensive Rasch PCM analysis firmly establishes and verifies that the VLQ-C possesses robust structural validity, confirming it as a highly reliable and culturally resonant instrument for assessing valued living among the Chinese population.

Complementing the Rasch findings, polychoric CFA provided additional evidence for the structural validity of the VLQ-C. The two-factor structure with correlated matched items and additional correlated residuals, informed by modification indices, was theoretically justified by the scale’s original intent. Since valued living is assessed by evaluating and comparing the individual’s assignment of importance and his or her consistent actions, matching the items between the two constructs is justified. Furthermore, the correlated residuals likely reflect shared content and methodological overlap between conceptually related item pairs rather than deficiencies in the measurement model. Taken together, the Rasch and CFA findings provide converging evidence that the VLQ-C possesses a psychometrically sound response structure and latent construct representation within the present sample.

The final CFA model included four theoretically justified and statistically supported correlated residuals. These residual correlations represent shared variance between specific item pairs beyond the two latent factors (Importance and Consistency). Two correlated residuals were observed in both the Importance and Consistency domains. The first linked Work and Education/Training, a theoretically expected association given the close relationship between education, career development, and vocational aspirations. Within the ACT framework, valued living reflects coherent actions across personally meaningful life domains; consequently, individuals who value education often view it as a pathway to meaningful work, thereby contributing to additional shared variance.

The second correlated residual involved Family and Marriage/Couple within the Consistency domain. These closely related interpersonal values jointly contribute to healthy family and intimate relationships. Consistent with ACT, behaviors that support marital relationships are closely intertwined with efforts to strengthen family life, making consistent actions in these domains naturally correlated. Within the Importance domain, a correlated residual was also identified between Family and Parenting. This relationship is conceptually expected, as parenting is a fundamental expression of family values. For parents, the importance assigned to parenting inherently reflects broader commitments to family, caregiving, and child development, leading to shared variance beyond the general valued living construct.

Overall, these correlated residuals reflect meaningful conceptual overlap between closely related life domains rather than data-driven modifications introduced solely to improve model fit. Their inclusion accounts for theoretically meaningful covariance between conceptually related domains while preserving the theoretical integrity of the original two-factor structure of the VLQ-C. Collectively, the Rasch and CFA findings provide complementary evidence supporting the proposed response structure and latent construct of the VLQ-C. Nevertheless, the elevated RMSEA suggests that some degree of model misfit remains, and further validation in independent samples is warranted.

There were a few limitations to consider in this study. First, the distributions of gender and psychiatric diagnoses in the study sample were not representative of the psychiatric patient population in China. Hence, the generalizability of the study findings may be affected. Moreover, this study recruited participants only from a single study site, which limits the representativeness of the sample and, in turn, undermines the generalizability of the findings. Second, criterion validity was not assessed in this study because no instrument measuring valued living has been translated and validated into Chinese. Hence, there is no gold standard instrument for comparison with the VLQ-C. Third, although the present study established construct validity through convergent validity, discriminant validity, Rasch analysis, and confirmatory factor analysis, known-groups validity was not examined. Future studies should evaluate whether the VLQ-C can discriminate between socio-demographic and clinical subgroups that are theoretically expected to differ in valued living, using larger and more clinically diverse samples.

While we examined gender DIF, a multigroup CFA invariance analysis was not performed due to the small, unbalanced sample (85 males, 195 females), which would likely yield unstable estimates. Using larger, more balanced samples to confirm VLQ-C measurement equivalence across genders will be useful in future studies.

Fourth, the Rasch PCM supports a 5-category VLQ-C, but the original face validity assessment used the 10-category format. As the item wording and instructions were unchanged and only adjacent categories were empirically collapsed, content and face validity can be considered to be are likely preserved. However, the revised format lacks formal evaluation. Future research should assess its comprehensibility, acceptability, and usability in independent samples. Finally, the choice of using the GAD-7 as a comparison tool for assessing discriminant validity may not be ideal, as the study sample consisted partly of patients with anxiety disorders, which may be related to the value of life that they regarded as important and consistent. This was reflected by the weak but significant inverse correlation coefficient between the VLQ-C domains and the total GAD-7.

Despite these limitations, the VLQ-C has demonstrated strong psychometric properties within both Classical Test Theory and Item Response Theory measurement frameworks, making it suitable for use among Chinese-speaking individuals with psychiatric conditions.

First, since valued living and committed action are key therapeutic objectives of Acceptance and Commitment Therapy (ACT), the VLQ-C serves as a culturally relevant tool for assessing treatment outcomes in Chinese-speaking populations. Notably, the analysis using the Rasch Partial Credit Model revealed that a simplified five-category response structure led to better category functioning, acceptable item fit, good reliability, and adequate targeting. This supports its use as a practical and psychometrically robust measure of valued living.

Second, the VLQ-C can be used to explore how valued living may act as a mediator or moderator in the relationship between mental illness, psychological flexibility, and quality of life. Such investigations could help clarify the mechanisms through which ACT and other values-based interventions improve psychological and functional outcomes.

Third, the VLQ-C may aid future research into the relationships between valued living and positive psychological constructs, such as psychological flexibility, resilience, well-being, and recovery. Future studies should also assess known-groups validity across socio-demographic and clinical subgroups and evaluate the VLQ-C’s responsiveness to therapeutic changes. This would further enhance its utility as both a research and clinical outcome measure.

## 5 Conclusion

This study successfully translated and culturally adapted the Valued Living Questionnaire into Chinese and demonstrated that the Chinese version (VLQ-C) has good psychometric properties among Chinese-speaking individuals with psychiatric illness. Evidence from both Classical Test Theory and Item Response Theory approaches supported its reliability and validity, including excellent face and content validity, good internal consistency, satisfactory test–retest reliability, and acceptable convergent and discriminant validity. Rasch Partial Credit Model analysis indicated that a 5-category response structure performed better than the original 10-category format, demonstrating improved threshold ordering, acceptable item fit, good targeting, and superior overall model performance. Polychoric confirmatory factor analysis further supported a two-factor item-paired structure with correlated residuals as the best-fitting representation of the construct. Although the findings support the proposed five-category response structure and two-factor construct, further replication in independent clinical and community samples, together with measurement invariance testing across demographic and diagnostic groups, is warranted before widespread implementation.
